# Assessing the effects of therapeutic combinations on SARS-CoV-2 infected patient outcomes: A big data approach

**DOI:** 10.1371/journal.pone.0282587

**Published:** 2023-03-09

**Authors:** Hamidreza Moradi, H. Timothy Bunnell, Bradley S. Price, Maryam Khodaverdi, Michael T. Vest, James Z. Porterfield, Alfred J. Anzalone, Susan L. Santangelo, Wesley Kimble, Jeremy Harper, William B. Hillegass, Sally L. Hodder, on behalf of the National COVID Cohort Collaborative (N3C) Consortium

**Affiliations:** 1 University of Mississippi Medical Center, Jackson, MS, United States of America; 2 Nemour’s Children’s Health, Wilmington, DE, United States of America; 3 West Virginia University, Morgantown, WV, United States of America; 4 West Virginia Clinical and Translational Science Institute, Morgantown, WV, United States of America; 5 Christiana Care Health System, Newark, DE, United States of America; 6 University of Kentucky, Lexington, KY, United States of America; 7 University of Nebraska Medical Center, Omaha, NE, United States of America; 8 Tufts University, Boston, MA, United States of America; 9 Owl Health Works LLC, Indianapolis, IN, United States of America; National Institute of Animal Biotechnology (NIAB), INDIA

## Abstract

**Background:**

The COVID-19 pandemic has demonstrated the need for efficient and comprehensive, simultaneous assessment of multiple combined novel therapies for viral infection across the range of illness severity. Randomized Controlled Trials (RCT) are the gold standard by which efficacy of therapeutic agents is demonstrated. However, they rarely are designed to assess treatment combinations across all relevant subgroups. A big data approach to analyzing real-world impacts of therapies may confirm or supplement RCT evidence to further assess effectiveness of therapeutic options for rapidly evolving diseases such as COVID-19.

**Methods:**

Gradient Boosted Decision Tree, Deep and Convolutional Neural Network classifiers were implemented and trained on the National COVID Cohort Collaborative (N3C) data repository to predict the patients’ outcome of death or discharge. Models leveraged the patients’ characteristics, the severity of COVID-19 at diagnosis, and the calculated proportion of days on different treatment combinations after diagnosis as features to predict the outcome. Then, the most accurate model is utilized by eXplainable Artificial Intelligence (XAI) algorithms to provide insights about the learned treatment combination impacts on the model’s final outcome prediction.

**Results:**

Gradient Boosted Decision Tree classifiers present the highest prediction accuracy in identifying patient outcomes with area under the receiver operator characteristic curve of 0.90 and accuracy of 0.81 for the outcomes of death or sufficient improvement to be discharged. The resulting model predicts the treatment combinations of anticoagulants and steroids are associated with the highest probability of improvement, followed by combined anticoagulants and targeted antivirals. In contrast, monotherapies of single drugs, including use of anticoagulants without steroid or antivirals are associated with poorer outcomes.

**Conclusions:**

This machine learning model by accurately predicting the mortality provides insights about the treatment combinations associated with clinical improvement in COVID-19 patients. Analysis of the model’s components suggests benefit to treatment with combination of steroids, antivirals, and anticoagulant medication. The approach also provides a framework for simultaneously evaluating multiple real-world therapeutic combinations in future research studies.

## Introduction

At the time of this writing, 8,029 completed or ongoing clinical trials for COVID-19 have been listed in ClinicalTrials.gov [1]. A majority of these trials are prospective randomized controlled trials (RCTs) or similarly designed clinical trials. These approaches offer the benefit of directly comparing therapeutic arms and control groups and can minimize bias. Notably, RCTs necessarily have inclusion and exclusion criteria that can limit the generalizability of the conclusions drawn from them. Further, RCTs, due to economic interests, required sample sizes, and the relative complexity of factorial designs, are rarely designed to explicitly address the optimal therapeutic combination(s) as a function of severity of illness.

Due to variable host-virus interactions, patients with SARS-CoV-2 infection may have a range of manifestations ranging from asymptomatic infection to critical illness [2]. Some studies of COVID-19 have described an initial viral stage of illness that can progress to a hyperinflammatory pulmonary stage, which can further evolve to a hypercoagulable phase or a late hyperinflammatory phase, as well as a chronic illness, referred to as the Post-Acute Sequelae of COVID-19 (PASC) or long-COVID [3]. Approaches to managing each of these phases are likely to require combinations of therapies directed at the respective underlying mechanisms and severity of illness. For instance, directly acting antiviral therapies would be anticipated to have the largest impact in the viral phase of the illness while anti-inflammatory treatments may be counterproductive as the body is mounting an antiviral immune response. By contrast, anti-inflammatory therapies would be expected to have the most beneficial effect in patients who have transitioned from the viral phase to a hyperinflammatory phase of illness. Antiviral agents may be less effective in this later phase. Especially in the early days of the pandemic, RCTs necessarily and largely evaluated individual therapeutic agents in critically ill patients, given the more favorable potential risk-benefit ratio. This approach may miss the impact of effective treatment combinations across the spectrum of COVID-19 illness severity.

Treatments have largely been studied individually in RCTs; for example, RCTs demonstrated that steroids benefit most patients requiring oxygen therapy [4], and the antiviral drug, Remdesivir, has been used successfully during the viral phase [5]. While one study recently reported benefit from the combination of Remdesivir and dexamethasone [6], data are lacking on the optimal combination of therapies for individual patients at various stages of the illness.

Review of patient outcomes from large, real-world data (RWD) sources offers the opportunity to assess the effect of therapies and their combinations not directly or adequately evaluated by RCTs, potentially augmenting our understanding of this increasingly complex therapeutic landscape [7]. Therefore, in this study, we explore the patient and treatment factors, particularly therapeutic agent combinations, associated with better outcomes using machine learning models (ML). The present study addresses key gaps in the extant literature by adopting ML models to evaluate the effect of therapeutic agents, singly and in combination, on patient outcomes using the large N3C cohort of patients.

## Methods

### Overall setting and study design

The National COVID Cohort Collaborative (N3C) is a high-granularity electronic health record (EHR) data repository containing harmonized, patient-level data from 72 sites across the United States (US). They are primarily tertiary care centers but also include data from health information exchanges and community hospitals. N3C data partners contribute data to N3C regularly. As of May 4, 2022 (Release 75), N3C contains data on more than 10 million patients, including more than 4.9 million COVID-19 SARS-CoV-2 infected persons.

N3C design, data ingestion and harmonization, and sampling approach have been detailed previously [8, 9]. In brief, N3C contributing sites provide the central repository EHR data, including demographics, healthcare visits, vital signs, medications, laboratory results, and diagnoses which are then harmonized into the Observational Medical Outcomes Partnership (OMOP) common data model. Participating sites submit EHR data on all patients with a positive SARS-CoV-2 lab test (Polymerase Chain Reaction, Antigen, or Antibody) or a COVID-19 diagnosis and a demographically matched comparison group of SARS-CoV-2 uninfected persons (1:2 matching positive: negative). For this study, we modify the N3C COVID-19 positivity definition [10] to exclude those with antibody-only positive results after December 10, 2020, the date when vaccinations became publicly available in the US [11].

To account for differences in data availability at the site level, we excluded sites with low medication reporting (<2 standard deviations below mean reporting for all sites). This approach excluded 17 of the 72 sites in N3C at the time of our data extraction.

Institutional Review Board (IRB) approval for this retrospective cohort study is obtained from the University of Mississippi Medical Center (IRB2020V0280, 3/31/2021), Johns Hopkins University (IRB00249128, 9/18/2020), Christiana Health (IRB604959, 5/07/2021), West Virginia University (IRB2012192778, 12/17/2020), University of Nebraska Medical Center (IRB050-21-EP, 2/9/2021), Nemour’s Children’s Health (IRB1700991, 2/17/2022), and Maine Medical Center (IRB1697848-2, 3/5/2021). Further approval by the N3C Data Access Committee (RP-504BA5) is granted that operates under the authority of the National Institute of Health IRB with Johns Hopkins University School of Medicine serving as the central IRB. A limited dataset was available for this project, however, zip codes were not used for the analyses described in the paper. No informed consent was obtained as the study utilizes a limited dataset.

### Cohort identification

For the purpose of this study, we selected COVID-positive patients with at least one day of hospitalization during the 28 days after their initial COVID-19 diagnosis. The cohort under study includes patients in the United States who tested positive for COVID-19 and were hospitalized between January 1, 2020, and July 1, 2021.

**Fig 1:**
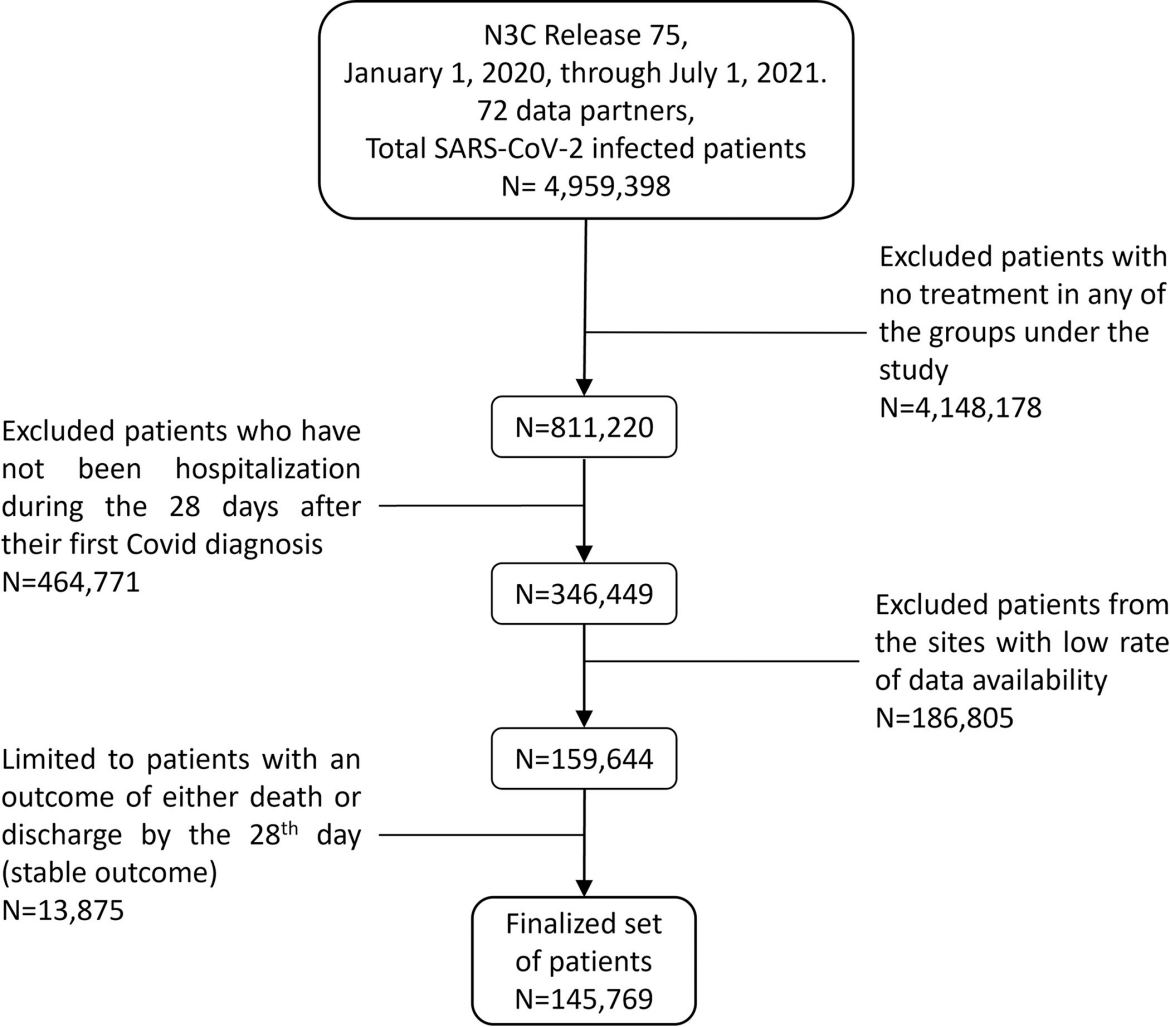
Information flow diagram for the cohort under the study.

Selection is then further limited to patients with an outcome of either death or discharge by the 28^th^ day (*n = 145*,*769*) after COVID-19 diagnosis. Patients with any other outcome at the end of the 28-day period are not considered as they are still being treated, and our interest is limited to those who have completed treatments [12–14]. This selected cohort is hereafter referred to as patients with a stable outcome, as treatment duration is completed and the final outcome of either death or discharge has been achieved. Fig 1 presents the information flow diagram for the final cohort under the study.

### Data extraction

Data were extracted on May 4, 2022 (N3C release 75) for the previously defined cohort with a stable outcome before July 1, 2021. The lag between the observation window cutoff and data extraction ensured that data from reporting sites was as complete as possible and placed the observation window before the rapid rise of the Delta variant. We developed concept sets for all conditions, drugs, and procedures used in this study, which include OMOP concept identifiers (derived from SNOMED CT, RxNorm, and other standardized vocabularies) contained with a patient’s EHR. Concept sets in use, available in Table 1, define computable phenotypes to programmatically identify patient health status at a point-in-time. All concept sets in use received review by three clinicians and one informatician during curation and implementation.

**Table 1:** Medications in each treatment category.

Class	Medication	Concept Sets
Anticoagulants (Coag)	Apixaban	259221776
	Betrixaban	568693141
	Dabigatran	23600781
	Enoxaparin	858278110
	Heparin	357794478
	Rivaroxaban	544420473
	Warfarin	441951686
Targeted Antivirals (ViralTrgt)	Remdesivir	719693192
	Nirmatrelvir/ritonavir (Paxlovid)	285332632
	Molnupiravir	643666235
Macrolide and Quinolone Antibiotics (BiotMQ)	Azithromycin	359938251
	Doxycycline	950251876
	Ciprofloxacin	369973585
	Moxifloxacin	609610642
	Gemifloxacin	382925247
	Delafloxacin	103404439
	Gatifloxacin	932126058
	Ofloxacin	931604126
	Norfloxacin	292248378
	Erythromycin	4697796
	Clarithromycin	4697796
	Levofloxacin	4697796
Spike Protein Monoclonal Antibodies (MonoSP)	Bamlanivimab	804283782
	Casirivimab/Imdevimab	204936358
	Etesevimab	985547691
	Sotrovimab	550646109
	Tixagevimab/Cilgavimab	809722294
	Bamlanivimab-Etesevimab combo	String search
	Bebtelovimab	String search
Steroids Preparations (Ster)	Dexamethasone	213873961
	Hydrocortisone	932266800, 422007021
	Methylprednisolone	640520004
	Prednisone	783588396
Monoclonal Antibody Immunomodulators (MonoI)	Tocilizumab	276204116
	Baricitinib	394764748
	Tofacitinib	391595378
	Sarilumab	807728943
Unproven Antiviral Therapies (ViralUnp)	Hydroxychloroquine	807281242
	Chloroquine	818210864
	Ivermectin	980395214
	Lopinavir/ritonavir	165611849
	Tenofovir	563211602, 568417090
	Interferon	359012050, 531467540
Miscellaneous (Misc)	Vitamin D	689338842
	Fluvoxamine	424477820

### Feature engineering

For the identified cohort, we have considered demographics, body mass index (BMI), comorbidities [15], treatment with pressors, the quarter of COVID-19 diagnosis, patient severity at the time of diagnosis, and prescribed treatments as input features for model development.

To measure patient severity, we used an Ordinal Scale (OS) developed for use with EHR data [16]. Specifically, this was a 6-point ordinal scale assigned with odd integers from 1 to 11, devised explicitly for patients diagnosed with COVID-19 based on discrete EHR data elements. In this context, a level of 1 represents an outpatient or patient discharged from the hospital, level 3 indicates hospitalization, while being hospitalized on Oxygen or Mechanical Ventilator is an indicator of levels 5 and 7, respectively, with level 9 representing patients hospitalized on ECMO and level 11 representing death.

**Fig 2:**
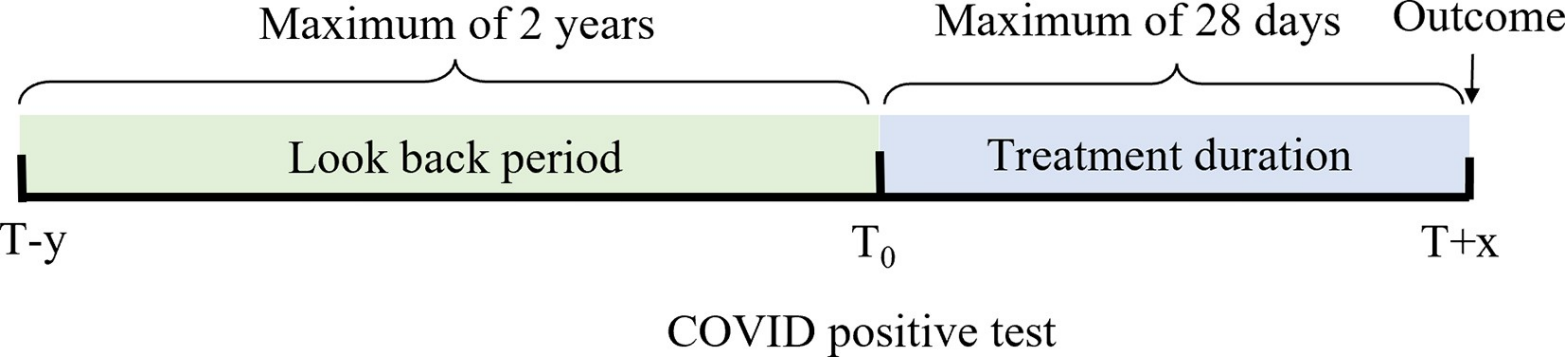
Time windows for treatment, comorbidities, and outcome.

Fig 2 shows the lookback period used for determining the patient’s comorbidities in green with a minimum of 2 years, while highlighted in blue are the considered treatments’ duration within up to 28 days after the diagnosis, followed by the recorded patient’s outcome as of the last day of treatment.

Prescribed therapeutics on each day after the diagnosis were categorized and considered in eight distinct groups, defined as anticoagulants (Coag), steroid preparations (Ster), unproven antiviral therapies (ViralUnp), targeted antivirals (ViralTrgt), spike protein monoclonal antibodies (MonoSP), monoclonal antibody Immunomodulators (MonoI), macrolide and quinolone antibiotics (BiotMQ), and a miscellaneous treatments (Misc) category that included other treatments presumed to be administered for treatment of COVID-19. Medications in each category are shown in Table 1.

The model considered the proportion of days on treatment combinations, any direct correlations between the treatment values and duration of treatment are removed, preventing the ML algorithm from leveraging this information directly for prediction. By using the proportion of days on treatment combinations, the modeling algorithm is forced to find the effect of different treatment distributions rather than attributing days on treatments to the outcome of interest.

### Modeling

We implemented three models to predict the final patient outcomes at the end of the 28-day observation window. The first was a Gradient Boosted Decision Tree (GBDT) classifier based on an additive model that tunes a weak learner into a strong one by training on residuals in boosting rounds; GBDT combines the results of previous learners along the way, thus learning from the errors of previous iterations to improve accuracy [17]. Two Neural Network models were also implemented, the first was based on a Deep fully-connected Neural Network (DNN) with a self-attention mechanism to increase the attention of the model to key features. The second was a multi-layer Convolutional Neural Network (CNN), convolving over the features to provide levels of generalization and extract treatment patterns and their effects. For the CNN model, multiple convolution structures based on VGG-16 [18], Inception [19], and DenseNet [20] blocks were evaluated and results for the best model is reported.

For the ML models, the input features considered as predictors of outcome are demographics, BMI, quarter of diagnosis, comorbidities, the severity of the patient at the time of diagnosis, being treated with pressors, and prescribed treatment combinations after diagnosis. Due to the sensitivity of ML models to hyper-parameters and to make the study repeatable, we used HyperOpt [21], an open-source Bayesian optimization library, to increase the model’s Area Under receiver operating Characteristic (AUC) curve by fine-tuning the parameters. Hyper-parameter tuning is performed on stratified random train, validation, and test splits of 60%, 20%, and 20% respectively, with random over-sampling of the training dataset using the SMOTE [22] library to address the data imbalance in the training set. Then, given the discovered hyper-parameters, model evaluation is conducted by 5-fold cross-validation to report the models’ AUC and accuracy.

### Model interpretability

Generally, machine learning models are considered black-box procedures, with limited insights and interpretability other than outcome prediction. However, recent years have seen many improvements in the ability to generate robust and interpretable insights from complex ML models [23]. Use of SHapley Additive exPlanation (SHAP) [24] values as an eXplainable Artificial Intelligence (XAI) algorithm can provide insightful interpretations of a complex machine learning model with high accuracy and robustness, similar to human interpretations.

The generated SHAP values for input features of ML models can be used to characterize the effect of the inputs on the final model’s prediction. In this study, to communicate the effects of treatment combinations as features of patients’ outcomes, we first trained an accurate ML model on the patients’ data. Then, the trained model is utilized for generating the SHAP values of input features, providing insights into the features’ importance on the probability of a patient discharge prediction. For the analysis, a feature has a positive impact if the feature increases the probability of the discharge prediction, while the negative impact of a feature translates to a decrease in the probability of discharge prediction.

After model hyper-parameter optimization, training, and evaluation, the model was retrained on the entire dataset, using the same parameters, to learn all existing interactions within the dataset. Then for SHAP value calculations, two required inputs are generated, background samples as a base of comparisons and input samples for evaluation of the effects. Following SHAP’s best practices for calculating the required background samples in large datasets, we applied the K-Nearest Neighbor clustering algorithm (K = 50) to patients in each class of outcome (death and discharge), providing us with a total of 100 cluster centroids to be used for the SHAP analysis.

For input samples, we noticed, however, that in a highly imbalanced dataset, averaging the SHAP values for each feature to provide a holistic view of the effect can be biased by the class containing the larger sample size (which in this case was discharge), diminishing the impact of learned interactions within the smaller set (defined by death as the outcome). To overcome any unwanted effects that data imbalance may pose on the results, we used 1:1 matched sets of patients, matching on the demographics (age, sex, race, ethnicity), BMI, comorbidities (specified in Table 2, under comorbidities), quarter of the year, pressor status (presence or absence), and OS level at diagnosis as inputs for SHAP calculation, resulting in a more balanced set of patients, preserving the effects and discriminating factors learned from the smaller set.

**Table 2:** Patients’ characteristics.

Characteristics	n = 145,769
Gender (%)	
Female	71,891 (49.3)
Male	73,855 (50.7)
Other/ Unknown	<25 (0.0)
Age (mean (SD))	59.2 y (19.5)
Race (%)	
Asian	5,305 (3.6)
Black	32,467 (22.3)
Native Haw./Pac. Islander	318 (0.2)
White	75,658 (51.9)
Other/ Unknown	30,883 (21.2)
Ethnicity (%)	
Hispanic/Latino	29,419 (20.2)
Not Hispanic/Latino	104,200 (71.5)
Other/ Unknown	12,068 (8.3)
OS at day 1 (%)	
OS 1—outpatient	35,328 (24.2)
OS 3—hospitalized	94,601 (64.9)
OS 5—hospitalized on Oxygen	9,326 (6.4)
OS 7—hospitalized on Mechanical Ventilator	6,252 (4.3)
OS 9—hospitalized on ECMO	262 (0.2)
OS 11—death	0 (0)
BMI (mean (SD))	31.0 (7.1)
Comorbidities (%)	
Hypertension	92,767 (63.6)
Diabetes Mellitus	33,808 (23.2)
Myocardial Infarction	19,197 (13.2)
Congestive Heart Failure	31,629 (21.7)
Peripheral Vascular Disease	24,011 (16.5)
Stroke	24,168 (16.6)
Dementia	13,037 (8.9)
Chronic Pulmonary Disease	40,545 (27.8)
Rheumatologic Disease	8,998 (6.2)
Mild Liver Disease	15,014 (10.3)
Severe Liver Disease	4,972 (3.4)
Upper GI bleed	4,791 (3.3)
Renal Disease	33,848 (23.2)
Peptic Ulcer Disease	4,496 (3.1)
Paralysis	5,116 (3.5)
Cancer	14,397 (9.9)
Diabetes with chronic complications	27,186 (18.7)
Metastatic solid tumor	5,376 (3.7)
HIV/AIDS	1,527 (1.0)
Quarter of Diagnosis (%)	
Jan-Mar 2020	6,956 (4.8)
Apr-Jun 2020	28,837 (19.8)
Jul-Sep 2020	17,111 (11.7)
Oct-Dec 2020	44,210 (30.3)
Jan-Mar 2021	33,468 (23.0)
Apr-Jun 2021	15,173 (10.4)
Outcomes (%, IQR)	
Discharged	128,063 (87.9, 8)
Death	17,706 (12.1, 13)

## Results

### Study population

The dataset included 145,769 hospitalized patients (Table 2). Most patients (128,063; 87.9%) were discharged alive from the hospital within 28 days of COVID-19 diagnosis while the remaining 17,706 (12.1%) were deceased. Although 24.2% of patients were not hospitalized on day 1 of their diagnosis (OS level 1), they subsequently were hospitalized after day 1 as this study assessed only hospitalized patients.

### Prescribed treatment combinations

**Fig 3:**
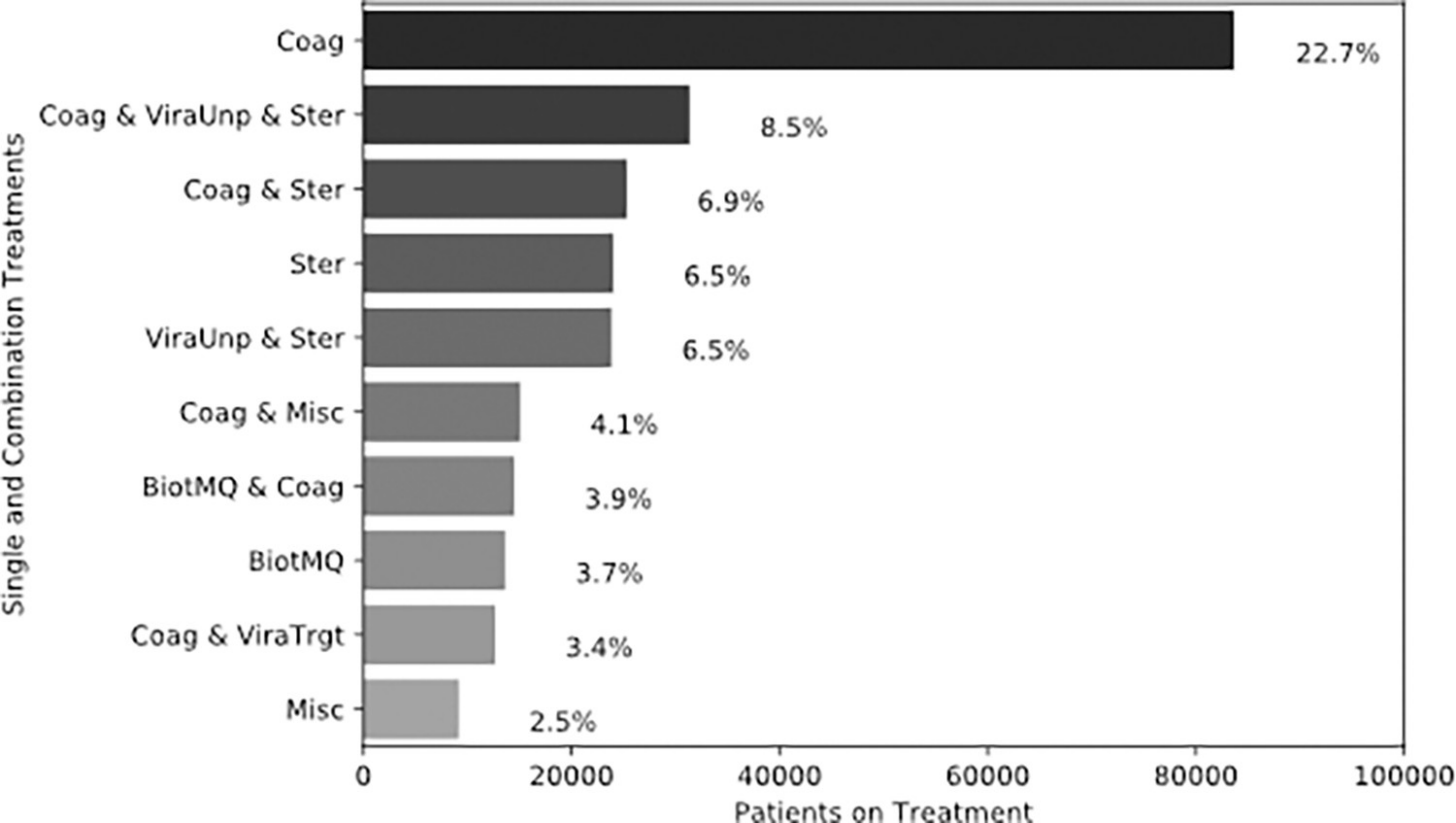
Top 10 prescribed treatments to patients.

Among single agent treatments, anticoagulants (Coag), steroids (Ster), and macrolide and quinolone antibiotics (BiotMQ) are the top three most commonly prescribed to patients; 22.7% (n = 83,665), 6.5% (n = 24,026), and 3.7% (n = 13,625), respectively (Fig 3). The three most frequent treatment combinations prescribed were: 1) anticoagulants and steroids with unproven antivirals (ViraUnp) 2) anticoagulants and steroids, and 3) steroids with unproven antivirals (ViraUnp) (Fig 3).

**Fig 4:**
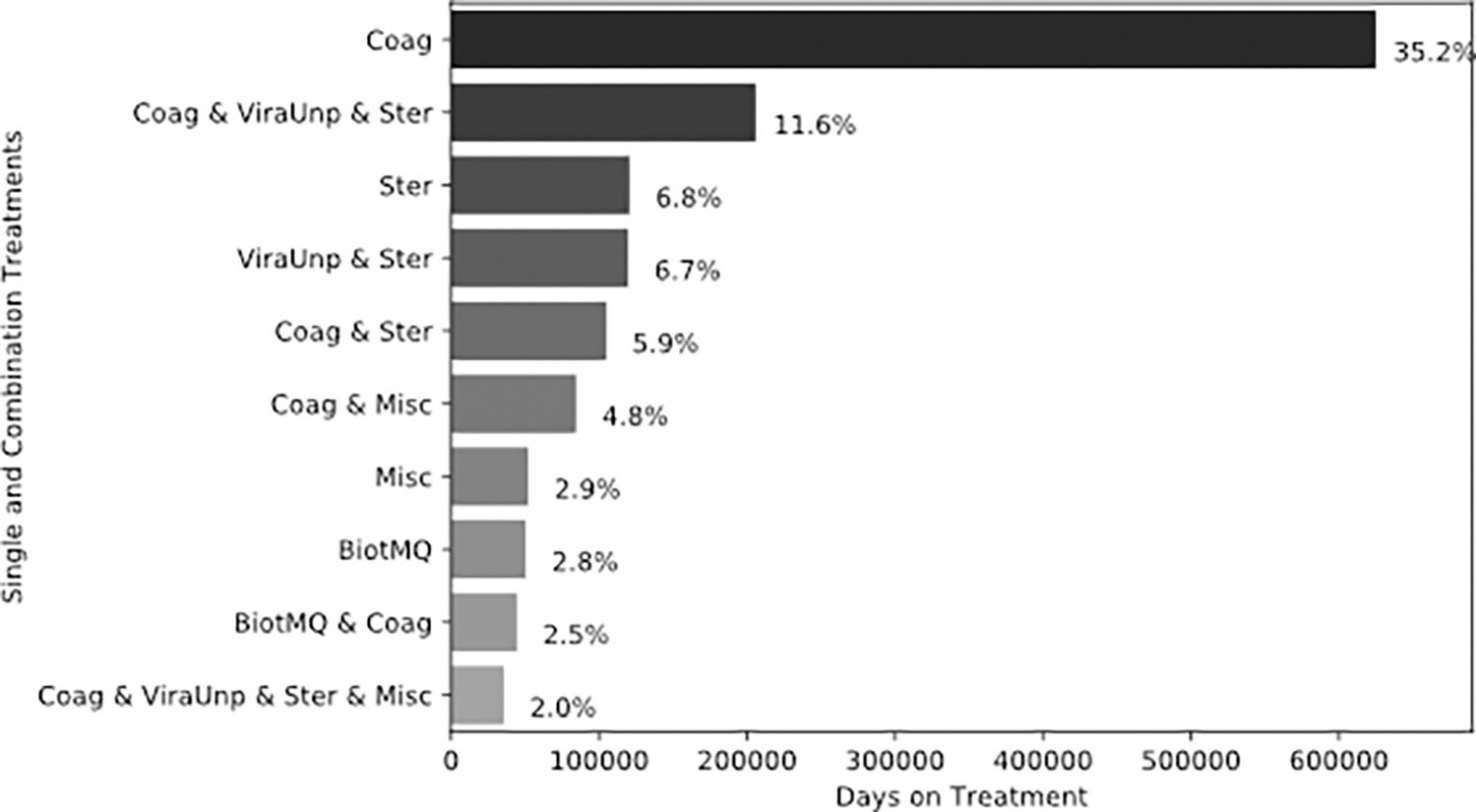
Top 10 treatments based on the number of days prescribed.

The top two prescribed treatments were also the treatments that patients received for the greatest number of days with 35.2% for anticoagulants and 11.6% for the combination therapy of anticoagulants with unproven antivirals (ViraUnp) and steroids. While steroids alone were the third most used therapeutic agent, patients spent roughly the same days on steroids in combination with anticoagulants (5.9%) and on steroid single therapy alone (6.8%). Fig 4 presents the top 10 therapeutics based on the cumulative number of days they were prescribed to patients.

### Model accuracy

**Fig 5:**
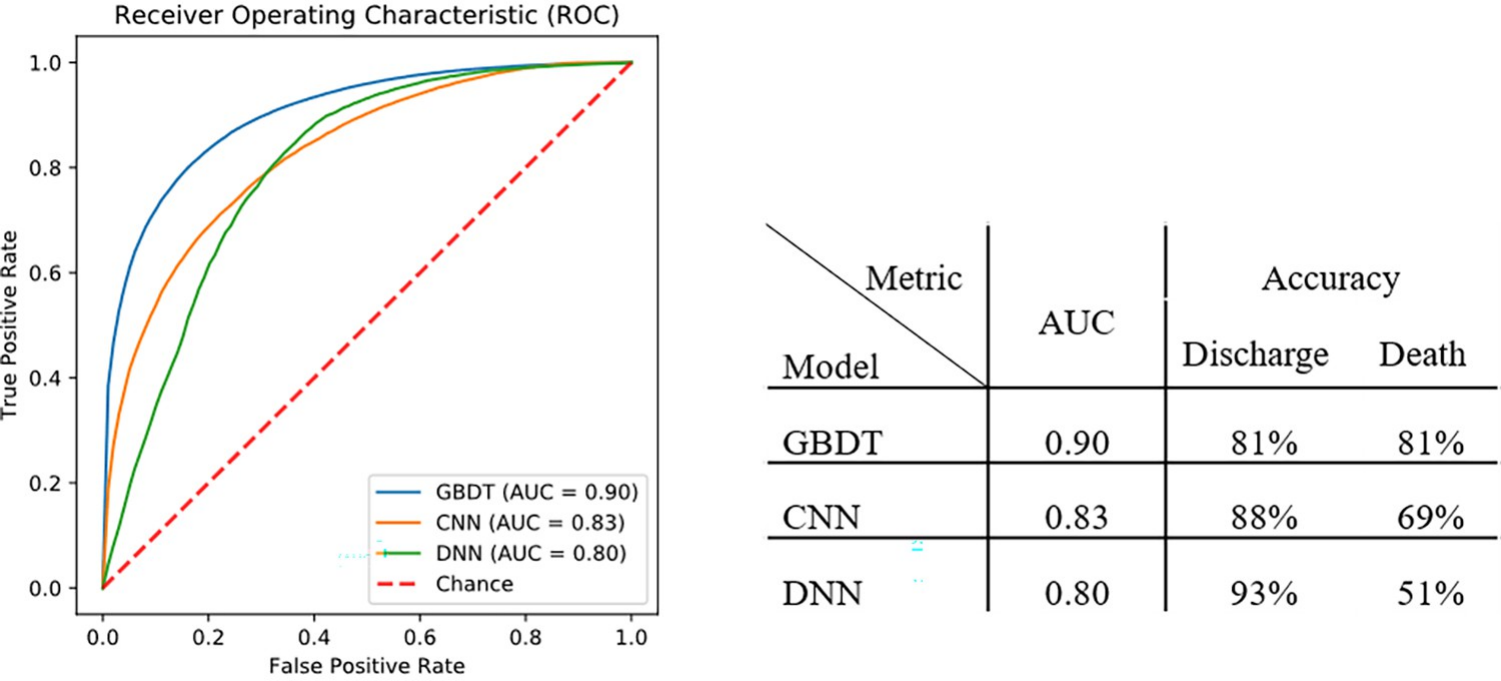
ML models performance in predicting patient outcomes.

Developing an ML model to leverage the aforementioned curated data and provide an accurate prediction, can be used not only as a predictive measure for taking therapeutic actions, but also as a means to evaluate the effect of patients’ characteristics and prescribed treatment combinations on the final patient outcomes. The devised models have been trained and evaluated using 5-fold cross-validation. Fig 5 shows the Receiver Operating Characteristic (ROC) curve, and accuracy of the models. Our results indicate that the Gradient Boosted Decision Tree (GBDT) classifier has superior (AUC = 0.90) and balanced accuracy (81% for both death and discharge classes) in identifying and discriminating patient outcomes compared to both Deep Neural Network (DNN) and Convolutional Neural Network (CNN) models.

### Feature importance

**Fig 6:**
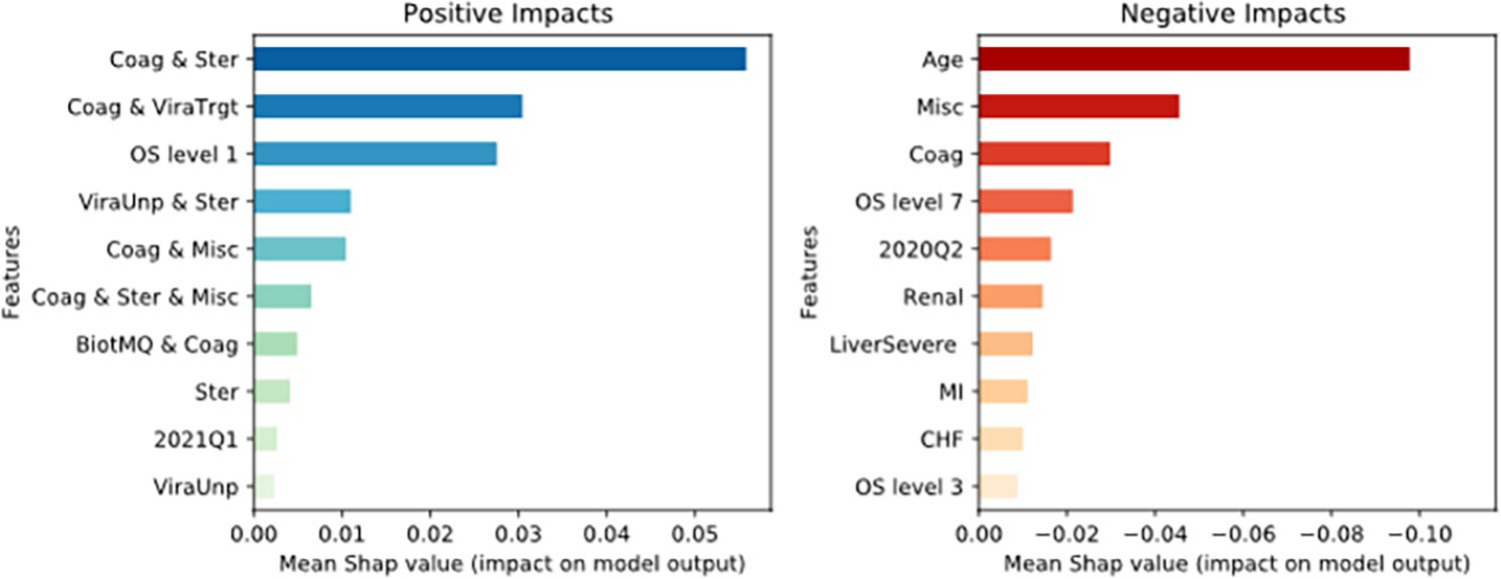
Top 10 features with the highest impact on final model prediction.

Given the accuracy and discriminative ability of the GBDT model, SHAP values were calculated to evaluate the impact of a feature on the model’s prediction. Specifically, positive SHAP values in this context indicate a positive impact on the predicted probability of classifying a patient as discharged while negative SHAP values indicate impact on the predicted probability of death. Fig 6 presents the top 10 features with the highest positive and negative impacts on the model’s predictive ability. It shows that six treatment combinations are among the top ten features with the highest positive impact underlining the importance of combination therapies; the steroid and anticoagulant combination provides the highest positive effect on model prediction. Monotherapies of both steroids and unproven antiviral therapies (ViralUnp) are ranked eighth and tenth after combination therapies. The other two features with high positive impact are COVID diagnosis in the first quarter of 2021 and OS severity level 1 (outpatient status) at the time of diagnosis. Among the features with the most negative impact, age is associated with the strongest negative impact on the model’s classification, followed by two of the single therapies: miscellaneous (Misc) and anticoagulants (Coag) alone. Among the comorbidities, renal disease (Renal), severe liver disease (LiverSevere), Myocardial Infarction (MI), and Congestive Heart Failure (CHF) are most highly associated with negative effects, in decreasing order of importance. In addition, OS levels 7 and 3 at the time of diagnosis are each associated with the negative outcome (death).

## Discussion

We developed accurate machine learning models with high accuracy for predicting death and discharge outcomes from COVID-19. By examing factors contributing to these predictions we can better understand the impact of treatment combinations on outcomes. Specifically, our findings suggest that combination therapy with different classes of drugs is more effective than therapy with only a single agent. These models also demonstrate that patient characteristics and comorbidities such as age, kidney, liver, heart disease, and severity of illness at diagnosis have a large impact on disease outcome, confirming previous literature [25–29]. Indeed, the models suggest that for COVID-19 outcomes, patient characteristics are not surprisingly often as influential as the treatments administered. Of note, pre-existing renal, liver, and heart diseases were strongly associated with poor prognosis. However, several combinations of treatments appear to be associated with better or worse outcomes. Specifically, our models support the efficacy of steroids, antiviral drugs, and anticoagulation while raising the possibility of harm from miscellaneous category therapies of vitamin D and fluvoxamine. Data from clinical trials of fluvoxamine in COVID-19 is mixed; however, our findings support guidelines recommending against its routine use at this time [30, 31]. Similarly, our negative findings regarding vitamin D are consistent with a clinical trial showing lack of efficacy of vitamin D in reducing the length of stay in hospitalized COVID-19 patients [32]. Steroids have a well established therapeutic benefit in COVID-19 patients requiring oxygen but have not been shown to benefit patients not requiring oxygen [33]. With electronic health record data, our models also observe the association between steroid treatment and higher likelihood of recovery among COVID-19 patients requiring oxygen therapy.

COVID-19 is associated with micro and macrovascular thrombosis [34–36], and COVID-19 patients have high risk of thrombotic complications such as pulmonary embolism. Therefore, various doses of anticoagulation have been proposed as part of standard COVID-19 treatment. Intermediate dose anticoagulation in ICU patients failed to show benefit [37]. However, among hospitalized patients not requiring ICU care, full dose anticoagulation has been reported to have benefits [38, 39]. If and when to use higher dose anticoagulation remains controversial [40, 41]. Our models suggest the possibility of benefit from the addition of anticoagulation to steroids. The combination of two potentially beneficial therapies, steroids and anticoagulants, being associated with an increased likelihood of recovery may reinforce the need to consider therapeutic combinations when attempting to define the optimal treatment of COVID-19.

The association with poor outcome of use of anticoagulants alone without steroids or antivirals is intriguing. Perhaps the use of anticoagulation alone is a marker for patients who were not treated aggressively for COVID-19 or who had comorbidities such as poorly controlled diabetes which might cause clinicians to withhold steroids or renal/hepatic failure that might give clinicians pause regarding the use of Remdesivir. However, this hypothesis cannot be tested in our dataset.

Many experts have suggested that combining steroids with antivirals may be beneficial because of the potential immunosuppressive effect of steroids [42, 43]. We expected to see a benefit of the combination of steroids and antivirals with efficacy against COVID-19. However, the overall positive effect of steroids combined with antivirals of unproven efficacy was surprising. It may be that the decision to use the combination of steroids/antiviral drug before proven antiviral drugs were available may have been a marker of other aspects of care (for example, excellent supportive care such as proning) that may have been associated with better outcomes. Since many patients in the dataset were treated before the availability of proven antivirals with efficacy against COVID, this may have led to an association between use of unproven (and likely ineffective) antivirals and reduced mortality.

Due to anti-inflammatory effects and proposed antiviral effects, macrolides were applied as possibly effective treatments for COVID-19 early in the pandemic [44]. Similarly, fluoroquinolone antibiotics were also suggested as COVID-19 treatments [45]. This was the rationale for including the antibiotics in our analysis. As enthusiasm for use of these medications for specific treatment of COVID-19 per se has declined, the positive associations found by our machine learning algorithms are perhaps unexpected. Severely ill COVID-19 patients are known to be at high risk of secondary infections [46]. It is possible that macrolides and quinolones treated secondary infections or prevented the development of such infections. Alternatively, the association between antibiotics treatment and improved outcome may be confounded by serving as a marker of more aggressive treatment. Further study of the mechanisms responsible for this association are needed.

While this study demonstrates a generally applicable machine learning model (ML) approach to explore treatment factors, particularly therapeutic agent combinations for COVID-19, ML models have been successfully applied to other aspects of the COVID-19 pandemic. More specifically for COVID-19, ML models have been developed and validated to predict the outcomes of COVID-19 patients using metrics collected at the time of admission [47]. Another study using ML evaluated risk factors associated with increased mortality for COVID-19 patients [48]. ML has also been used to show the predictive effect of comorbidities and risk factors on progression of illness in COVID-19 patients [49, 50]. ML models generally demonstrate improved prediction of patient outcomes when compared to conventional statistical approaches [51–53].

Our study has several limitations. First, information on the doses of medications used is not available in the dataset. Similarly, the impact of steroid dose is unknown. However, the results of our study support the need for clinical trials to explore the efficacy of different doses of therapeutic combinations and single therapies. An additional limitation is that we have no knowledge as to why clinicians choose to administer or not administer certain therapeutic agents. Patients with treatment limitations, such as DNR orders, are more likely to die than those without such limitations [54]. It is possible that such care limitations or contraindications, especially early in the pandemic, influenced the decision to use or not use certain treatments. It is also possible that some patients were incidentally positive for COVID-19 but hospitalized for other serious illnesses, although this cannot be determined from the database. Another limitation of our study is the lack of full control over the diagnosis criteria that treating clinicians used and the possibility of false negatives or false positives, however, we followed the best practices provided by the NIH experts to define inclusion criteria for COVID-19 positivity.

## Conclusions

Machine learning algorithms can predict mortality in hospitalized COVID-19 patients with a high degree of accuracy. Future work may allow use of such algorithms to identify high risk patients needing more aggressive therapies. In the meantime, our analyses of a large multicenter cohort of COVID-19 patients using machine learning algorithms supports use of steroids, anti-virals, and anticoagulant medications in combination. Further study is needed on the associations of macrolide and fluoroquinolone antibiotics with survival in COVID-19. In addition to the beneficial observed effects of specific treatments and, in particular, their combinations, patient characteristics such as age and comorbidities are strong predictors of increased likelihood of death as expected, perhaps serving as negative controls suggesting validity of the models. More generally, this study demonstrates use of a machine learning model (ML) approach to explore treatment factors, particularly therapeutic agent combinations, associated with outcomes across comorbidity profiles and initial severity of illness. It potentially provides useful evidence, particularly with regard to therapeutic combinations, to supplement evidence from RCTs.

## Supporting information

S1 File (DOCX)

S1 Fig Top 4 therapeutics with a positive effect on patients’ outcome: Overall and stratified cohorts. (TIF)

S2 Fig Top 4 therapeutics with a positive effect on patients’ outcome: Overall and stratified cohorts. (TIF)
