# Supplementary material for: Assessing the effects of therapeutic combinations on SARS-CoV-2 infected patient outcomes: A big data approach
Source: PLoS One. 2023 Mar 9;18(3):e0282587. doi: 10.1371/journal.pone.0282587 (PMC9997963; doi:10.1371/journal.pone.0282587)
Supplement: S1 File — (DOCX) [file pone.0282587.s001.docx]

The patients were not all at the same stage of disease at the time of admission. Patients under the study could be at any of the first 5 initial levels of severity at the time of admission (OS-Level of 1, 3,5, 7, or 9). However, it should be noted that 75.8% of patients were hospitalized on the days of diagnosis, while the remaining were hospitalized at some point during the 28 days after diagnosis (with a median of 2 days).

For sensitivity analysis, we evaluated the exact same methodologies where patients are stratified by their initial OS level. This means, developing a model for each cohort with patients in one of the severity levels at the time of admission. Figs S1 and S2 present the results for the therapeutics with positive and negative effects on the overall and subsets of the same cohort with different initial severity levels.

We can observe that the top four therapeutics with positive and negative effects are exactly the same for the overall cohort compared to the subsets of the cohort stratified by the initial OS level. This will further confirm our findings and the consistency of our results.
