# Supplementary figures and images for: Assessing the effects of therapeutic combinations on SARS-CoV-2 infected patient outcomes: A big data approach
Source: PLoS One. 2023 Mar 9;18(3):e0282587. doi: 10.1371/journal.pone.0282587 (PMC9997963; doi:10.1371/journal.pone.0282587)

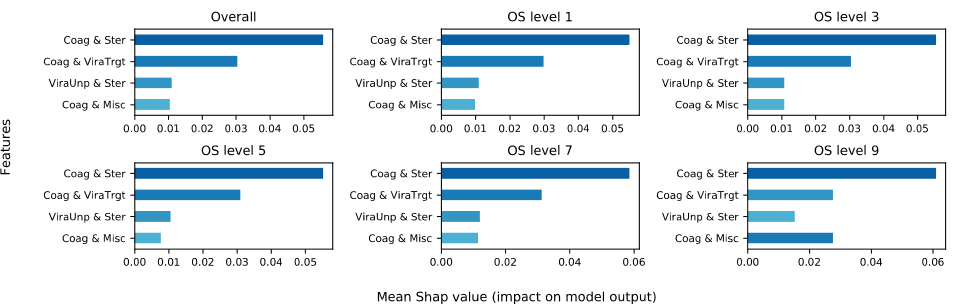

Supplement: S1 Fig — (TIF) [file pone.0282587.s002.tif]

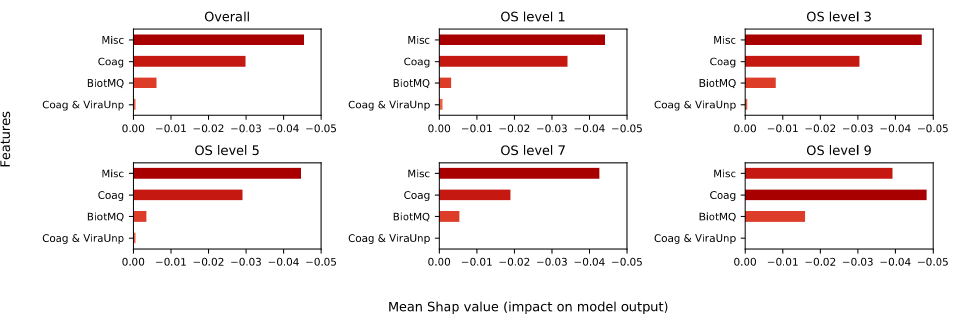

Supplement: S2 Fig — (TIF) [file pone.0282587.s003.tif]
